# Poly(l-Lactic Acid)-co-poly(Butylene Adipate) New Block Copolymers for the Preparation of Drug-Loaded Long Acting Injectable Microparticles

**DOI:** 10.3390/pharmaceutics13070930

**Published:** 2021-06-23

**Authors:** Vasiliki Karava, Aggeliki Siamidi, Marilena Vlachou, Evi Christodoulou, Nikolaos D. Bikiaris, Alexandra Zamboulis, Margaritis Kostoglou, Eleni Gounari, Panagiotis Barmpalexis

**Affiliations:** 1 Department of Pharmacy, Section of Pharmaceutical Technology, Zografou Campus, National and Kapodistrian University of Athens, 15784 Athens, Greece; vaso.karava111@gmail.com (V.K.); asiamidi@pharm.uoa.gr (A.S.); 2 Department of Chemistry, Laboratory of Polymer Chemistry and Technology, Aristotle University of Thessaloniki, 54124 Thessaloniki, Greece; evicius@gmail.com (E.C.); nbikiaris@gmail.com (N.D.B.); azampouli@chem.auth.gr (A.Z.); 3 Laboratory of Chemical and Environmental Technology, Aristotle University of Thessaloniki, 54124 Thessaloniki, Greece; kostoglu@chem.auth.gr; 4 Biohellenika Biotechnology Company, Leoforos Georgikis Scholis 65, 57001 Thessaloniki, Greece; egounari@biohellenika.gr; 5 Department of Biochemistry, School of Medicine, Faculty of Health Sciences, Aristotle University of Thessaloniki, 54124 Thessaloniki, Greece; 6 Department of Pharmaceutical Technology, School of Pharmacy, Aristotle University of Thessaloniki, 54124 Thessaloniki, Greece; pbarmp@pharm.auth.gr

**Keywords:** long acting injectables, poly(l-lactic acid), poly(butylene adipate), block copolymers, aripiprazole, microparticles, sustained release

## Abstract

The present study evaluates the use of newly synthesized poly(l-lactic acid)-co-poly(butylene adipate) (PLA/PBAd) block copolymers as microcarriers for the preparation of aripiprazole (ARI)-loaded long acting injectable (LAI) formulations. The effect of various PLA to PBAd ratios (95/5, 90/10, 75/25 and 50/50 *w*/*w*) on the enzymatic hydrolysis of the copolymers showed increasing erosion rates by increasing the PBAd content, while cytotoxicity studies revealed non-toxicity for all prepared biomaterials. SEM images showed the formation of well-shaped, spherical MPs with a smooth exterior surface and no particle’s agglomeration, while DSC and pXRD data revealed that the presence of PBAd in the copolymers favors the amorphization of ARI. FTIR spectroscopy showed the formation of new ester bonds between the PLA and PBAd parts, while analysis of the MP formulations showed no molecular drug–polyester matrix interactions. In vitro dissolution studies suggested a highly tunable biphasic extended release, for up to 30 days, indicating the potential of the synthesized copolymers to act as promising LAI formulations, which will maintain a continuous therapeutic level for an extended time period. Lastly, several empirical and mechanistic models were also tested, with respect to their ability to fit the experimental release data.

## 1. Introduction

In the past few decades synthetic polymers that degrade under physiological conditions (i.e., biodegradable polymers) have become increasingly common in medical and pharmaceutical applications [1,2,3]. Especially, in the case of particulate drug formulations (such as nano- or microparticles) an increasing number of polymeric materials, and especially polyesters, have been introduced and implemented for drug delivery [4,5,6]. Amongst them, the preparation of long-acting injectable (LAI) formulations is probably the most intensively studied application for such polyester-based systems. In general, LAIs’ are being utilized to reduce drug administration frequency, resulting to higher patient compliance. Compared to other formulations, LAIs can provide a prolonged and constant therapeutic effect, enhance the biological half-life of drugs, improve bioavailability and protect the active pharmaceutical ingredients (APIs) against harsh environmental conditions [7,8,9]. In addition, compared to other materials, such as lipids, polyesters can be customized more easily in order to adapt to any specific type of drug [4]. However, despite the numerous scientific reports, the fact that only about 20 different LAI products are available in the market, suggests that the design of such drug formulations is a rather difficult task [10].

In this context, perhaps the most widely explored polyester used is poly(l-lactide) (PLA) and its copolymer with glycolic acid (i.e., poly (lactic-co-glycolic acid), PLGA) [11,12]. In general, PLA or PLGA LAI formulations have been investigated as suitable matrix/carriers to deliver a variety of APIs, including small molecules, peptides and proteins for periods ranging from one week to several months [13,14,15,16,17,18,19]. In the last two decades several PLA or PLGA-based products have been brought into the market [11,20,21]. The characteristics of the polymer, such as molecular weight (MW), copolymer composition, terminal groups functionality and glass-transition temperature (T_g_) are the key factors affecting its biodegradability and, hence, release kinetics. However, despite their inherent flexibility, PLA and PLGA-based LAIs face a number of challenges, including initial burst release, enhanced lag-time, incomplete drug dissolution and poor drug stability during both production and storage [10,11]. In an attempt to overcome these limitations, a wide range of suitable biodegradable polymers, including poly-ε-caprolactone, polyorthoesters, polydioxanones, polyphosphazenes, polyanhydrides, poly(acyanoacrylates), polyiminocarbonates, polyoxalates and polyurethanes, have been proposed as alternatives. Among them, poly(alkylene adipate) derivatives were only recently introduced showing promising results [22,23].

In general, poly(alkylene adipate)s, derived from dicarboxylic acids and different aliphatic diols, such as poly(ethylene adipate) (PEAd)), poly(propylene adipate) (PPAd) and poly(butylene adipate) (PBAd), seem to be a promising PLA or PLGA substitute in terms of ecological and economic (balance of cost–benefit) factors [24,25,26,27]. In a recently published attempt, the use of poly(alkylene adipate)s, as sole matrix/carriers for the preparation of drug LAI microparticle formulations showed promising results, in terms of efficacy, although incomplete drug dissolution was recorded in addition to rather short sustained action (a plateau was reached in dissolution at 3 days) [22]. These results indicate that a certain amount of tunning is needed in order for this type of polyesters to be suitable for LAI formulations. Similar results were also obtained from another study, where the combination of poly(butylene adipate) (PBAd) with PLA in the form of a physical blend was utilized in order to prepare novel electrospun nanofibrous matrices for the sustained delivery of the immunomodulatory drug, teriflunomide [23]. In this study, a controlled release pattern of the drug was achieved and varied analogous to the proportion of the PBAd and the drug content.

**Figure 1:**
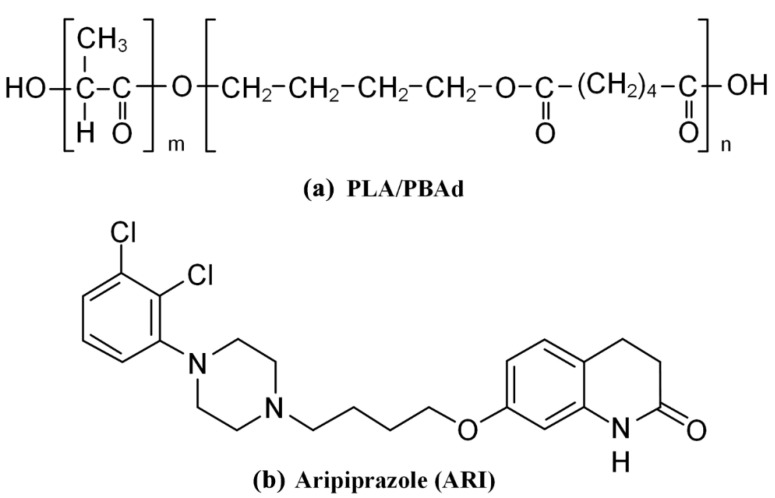
Chemical structures of (**a**) PLA/PBAd copolymers and (**b**) aripiprazole (ARI).

In view of these findings, we recently published a study on the synthesis of a new block copolymer with enhanced physicochemical and mechanical performance, based on butylene adipate segments [28]. Specifically, block copolymers of PBAd combined with PLA (Figure 1a) were synthesized via a two stage polycondensation and analyzed in regard to thermal and mechanical properties. The results showed that the continuity of the two polymers throughout the copolymer volume and the semicrystalline morphology were both easily tuned by either the preparation method conditions and the ratio of PBAd to PLA to the drug. Based on these features it can be assumed that the new prepared block copolymer may be a promising candidate for the preparation of drug-loaded LAI microparticles (MPs).

In the present study the use of the recently synthesized PLA/PBAd block copolymer was evaluated as a suitable LAI carrier. Aripiprazole (ARI, Figure 1b), a second-generation antipsychotic, is used as a model drug, which according to the FDA, is one of the several antipsychotic drugs marketed as a LAI formulation (please see Abilify Maintena^®^), which, however, presents a long initial lag-time (this is why oral ARI is simultaneously given for 14 consecutive days after the initial LAI injection) and is administrated rather frequently (i.e., once per month) [29,30,31]. Hence, within the set of the present study, after the initial evaluation of the biodegradation and the cytotoxicity profile of the neat PLA/PBAd block copolymers, ARI-loaded PLA/PBAd MPs were prepared via the emulsification/solvent evaporation method and thoroughly evaluated in terms of physicochemical and pharmacotechnical properties.

## 2. Materials and Methods

### 2.1. Materials

ARI (7-(4-[4-(2,3-Dichlorophenyl) piperazin-1-yl]butoxy)-3,4-dihydroquinolin-2(1*H*)-one) form III crystals were kindly donated by Pharmathen S.A. (Athens, Greece). Adipic acid (ACS reagent, ≥99.0%), 1,4-butanediol (99%), tetrabutyl titanate (TBT) (97%) and the Tin(II) 2-ethylhexanoate (TEH) (96%) catalysts were obtained from the Sigma-Aldrich (Saint Louis, MO, USA). l-Lactide (98%) and (*S*,*S*)-3,6-dimethyl-1,4-dioxane-2,5-dione were purchased from Alfa Aesar Chemicals (Kandel, Germany). *Rhizopus delemar* and *Pseudomonas cepacia* lipases were purchased from Fluka BioChemika, Steinheim, Germany. All other solvents and reagents used were of analytical or pharmaceutical grade and were used as received.

### 2.2. Synthesis of PBAd and PLA/PBAd Block Copolymers

PBAd and PLA/PBAd copolymers were prepared by the method we previously published [28]. Briefly, PBAd was prepared via a two-stage esterification and polycondensation. During the first stage (esterification), accurately weighed amounts of adipic acid and 1,4-butanediol, in a 1/1.1 molar ratio, were placed in a round-bottom flask and the polymerization mixture was degassed and purged with nitrogen several times, before heating to 180 °C under constant stirring and then gradually heating up to 220 °C over a period of three hours. After removal of the water formed, the nitrogen flow was stopped and 400 ppm of TBT (0.05 g mL^−1^ in toluene) was added to the mixture under high vacuum (5.0 Pa), in order to avoid excessive foaming. The temperature was then increased to 240 °C and the polycondensation reaction was carried out for another two hours.

After the preparation of the neat PBAd the PLA/PBAd copolymers were prepared via ring opening polymerization of l-lactide. Briefly, proper amounts of l-lactide and PBAd (corresponding to a final copolymer weight ratio of 95/5, 90/10, 75/25 and 50/50 *w*/*w* PLA to PBAd) were placed in round bottom flasks along with the THE (used as a catalyst at 400 ppm based on the l-lactide concentration). After nitrogen purging, the mixture was heated up to 200 °C and the reaction was initiated and carried out under constant mechanical stirring for one hour. The MW of the prepared copolymers was increased by heating up to 220 °C, under high vacuum (5.0 Pa) for 15 min. Then, the flasks were cooled to room temperature and the copolymers were purified by dissolving them in chloroform and precipitating in cold methanol twice, prior to using them. The precipitates were filtered and dried in a vacuum oven at 50 °C for 24 h. All samples were collected, placed in hermetically sealed vials, after purging with N_2_, and stored at 5 °C before further use.

### 2.3. Characterization of PLA/PBAd Block Copolymers

Following their preparation, the newly synthesized block copolymers were characterized in terms of biodegradation and cytotoxicity profiles, characteristics that are extremely significant when preparing drug LAI formulations. Results, in terms of structural characterization, MW, physical state, thermal properties and molecular mobility, are given in a previous study of ours (Table 1) [28].

#### 2.3.1. Enzymic Hydrolysis

PLA/PBAd enzymatic hydrolysis was performed based on a previously employed method [32]. Briefly, the neat PBAd and PLA and the PBAd/PLA copolymers were prepared in the form of films, using an OttoWeber Type PW 30 hydraulic press (Paul-Otto Weber GmbH, Remshalden, Germany). The films were placed in petri dishes and 5 mL of phosphate buffer solution (0.2 M, pH 7.4) was added, containing 0.09 mg/mL of *Rhizopus delemar* lipase and 0.01 mg/mL of *Pseudomonas cepacia* lipase. The petri dishes were kept at 37.0 ± 1.0 °C in an oven for twenty days, while the media were replaced every 24 h. After predetermined time intervals, the films were removed from the lipase solution, washed thoroughly with distilled water and dried at 40 °C in vacuo, until constant weight. Every measurement was repeated three times. The degree of enzymatic hydrolysis was estimated from the weight loss, as compared to the initial weight of the samples.

#### 2.3.2. Size-Exclusion Chromatography

The molecular weights of all samples after enzymatic hydrolysis were estimated by size-exclusion chromatography (SEC). The analysis was performed by means of SEC equipment consisting of a Waters 600 high pressure liquid chromatographic pump (Waters, Milford, MA, USA), Waters Ultrastyragel columns (HR-1, HR-2, HR-4 and HR-5) and a Shimadzu RID-10A refractive index detector (Shimadzu Corporation, Kyoto, Japan). Column calibration was performed using polystyrene standards (1–300 kg/mol in molecular weight). The concentration of the prepared solutions was 20 mg/1000 mL, the injection volume was 150 mL and the flow rate was 1 mL/min, operating at 60 °C.

#### 2.3.3. Cytotoxicity Studies

Human adipose-derived mesenchymal stem cells culture (hAMSCs): For the evaluation of neat polymer and copolymers cytotoxicity, hAMSCs were provided from Biohellenika S.A. (Thessaloniki, Greece) after adipose tissue isolation from healthy volunteer donors. Experimentally, after liposuction adipose tissue washed twice in PBS (phosphate buffered saline) (1X, pH 7.4) (BIOWEST, Nuaillé, France). Overnight digestion was performed with 5 mg collagenase type I (Sigma-Aldrich, Saint Louis, MO, USA) per 10 g of adipose tissue after overnight incubation. The mixture was filtered using a 70 μm cell strainer (CORNING, Glendale, AZ, USA) and centrifuged at 850× *g* for 10 min. The pellet was resuspended in Dulbecco’s modified Eagle’s medium (DMEM)(BIOWEST, Nuaillé, France) supplemented with 10% fetal bovine serum (FBS)(BIOWEST, Nuaillé, France) and 2% penicillin/streptomycin and plated in culture flasks for 72 h until cells’ adherence to the plastic surface (37 °C incubation with 5% CO2). The cell culture medium was replaced every 2–3 days until 80–90% confluence was reached. Cells were used in the experiments between passage 4 and 5. Every cells’ detachment was performed with 0.05% trypsin–EDTA (BIOWEST, Nuaillé, France).

Sterilization of the materials and cell seeding: All the materials were sterilized in gradually reduced ethanol concentrations (100%, 70% and 50% in ddH_2_O) and, after washing twice with ddH_2_O, were left to air dry for 5 h under sterile conditions. Fibrin glue was prepared after the blood sampling of a healthy volunteer donor. A total of 10 μL of fibrin glue per film were placed in the bottom of a 24-well plate and the materials were seeded using a sterile pincher from above by applying minimal manual pressure and were left to air dry overnight under sterile conditions.

hAMSCs were detached using trypsin–EDTA 1x in PBS. A total of 3.5 × 10^5^ cells were resuspended in the DMEM full medium and were subsequently placed above the films of each condition. A total of 3.5 × 10^5^ cells were also plated in a plastic surface without any material and used as a control group. Upon air drying for 4 h in the incubator 1 mL of the DMEM full medium was added per well for the culture initiation. After 48 h, the cytotoxic effect of the materials was determined with an MTT (3-[4,5-dimethylthiazol-2-yl]-2,5 diphenyl tetrazolium bromide) assay.

In vitro cytotoxicity assays: The MTT cell proliferation assay, which employs the reduction of tetrazolium salts by metabolically active cells for examining cellular viability, was used for in vitro cytotoxicity assessment (Trevigen, Gaithersburg, MD, USA 4890-025-K). After 48 h of coincubation with the formulations, the medium was removed and cells were washed once with PBS before adding fresh medium including the 1/10 MTT reagent (Sigma-Aldrich, Saint Louis, MI, USA). Upon the removal of the MTT, 1 mL/well of DMSO was introduced for one additional hour of incubation. The optical density of MTT formazan deposits was quantified by a spectrophotometer at a 570 nm and 630 nm wavelength (PerkinElmer, Boston, MA, USA). All experiments were conducted in triplicate.

### 2.4. Preparation of ARI MPs

ARI MPs were prepared using PLA and PBAd polymers and their copolymers using an emulsification/solvent evaporation method. Briefly, 250 mg of polymer (pure PLA, pure PBAd and copolymers) were initially dissolved in 5 mL of dichloromethane and stirred with a magnetic stirrer. Then 50 mg of ARI were added to the solution and sonicated for 1 min until complete dispersion. The aqueous phase (50 mL of deionized H_2_O and 50 mL of 1% *w/v* PVA solution) was then added to the dispersion phase, homogenized and left under stirring (1200 rpm), at room temperature, until the solvent was completely evaporated. When the microspheres were formed, they were separated from the rest of the solution by centrifugation at 4500 rpm for 10 min. Possible solvent or emulsifier residue was removed by three consecutive washes with deionized water. The microspheres were then freeze-dried in order to remove any water residue. For the preparation of non-drug loaded MPs, the same procedure as the one described above was followed without the addition of the API. All final samples were subsequently stored at 4 °C using hermetically sealed amber glass vails before further use.

### 2.5. Characterization of MPs

#### 2.5.1. Differential Scanning Calorimetry (DSC)

DSC studies were conducted, using a Perkin–Elmer, Pyris Diamond DSC. In brief, accurately weighed samples (5.0 ± 0.1 mg) of the raw materials (i.e., neat PLA, neat PBAd and neat ARI) and the PLA-ARI, PBAd-ARI and PLA/PBAd-ARI MPs were hermetically sealed in aluminum pans and placed in the DSC sample holder. Then the samples were heated from 25 to 180 °C with a heating rate of 20 °C/min and the various thermal events were recorded using the Pyris Diamond software. The melting points (T_melt_) were determined as the peak temperature and the glass-transition temperature (T_g_) was determined as the inflection point temperature, while the enthalpy of fusion (ΔH_f_) was determined as the integrated area of the heat flow curve in all cases. Nitrogen flow (50 mL/min) was applied in order to provide a constant thermal blanket within the DSC cell. The instrument was calibrated for temperature using high purity benzophenone, indium and tin, while the enthalpic response was calibrated using indium. All measurements were conducted in triplicate. The standard deviations of temperatures and enthalpies determined, in this work, were not higher than 1.0 °C and 3.0 J/g, respectively.

#### 2.5.2. Wide Angle Powder X-ray Diffractometry (pXRD)

pXRD patterns of the raw materials (i.e., neat PLA, neat PBAd and neat ARI) and the PLA-ARI, PBAd-ARI and PLA/PBAd-ARI MPs were recorded using an XRD-diffractometer (Rigaku-Miniflex II, Chalgrove, Oxford, UK) with a CuKα radiation for crystalline phase identification (λ = 0.15405 nm for CuKα). All samples were scanned from 5 to 50° with a scanning rate of 1 °/min.

#### 2.5.3. Scanning Electron Microscopy (SEM)

The morphology of the neat PLA, PLBAd and PLA/PBAd copolymers in the form of film before and after the enzymatic hydrolysis study and the ARI-loaded PLA, PBAD and PBAd MPs before and after the completion of the dissolution study was examined in a SEM system (JEOL JMS-840, JEOL USA Inc., Peabody, MA, USAmanufacturer, city, country). All samples (either in the form of thin films or MPs) were covered with carbon in order to provide good conductivity of the electron beam. All SEM images were collected with the following operating conditions: (1) accelerating voltage 20 kV, (2) probe current 45 nA and (3) counting time 60 s.

#### 2.5.4. Fourier-Transformed Infrared Spectroscopy (FTIR)

The chemical structure and the formation of molecular interactions in PLA/PBAd copolymers and the PLA-ARI, PBAd-ARI and PLA/PBAd-ARI MPs was elucidated by FTIR spectroscopy. FTIR spectra of the samples were received with an FTIR spectrophotometer (model FTIR-2000, Perkin Elmer, Dresden, Germany) using KBr discs (thickness of 500 μm). The spectra were collected in the range from 4000 to 400 cm^−1^ at a resolution of 2 cm^−1^ (total of 64 coadded scans) and were baseline corrected and converted into the absorbance mode.

#### 2.5.5. Yield, Encapsulation Efficiency and Drug Loading

MPs’ yield, drug loading and encapsulation efficiency (EE) were determined by applying the following equations:

Yield (%) = [weight of MPs]/[initial weight of polymers and ARI] × 100(1)

Drug loading (%) = [weight of ARI in MPs]/[total weight of MPs] × 100(2)

EE (%) = [weight of ARI in MPs]/[initial weight of ARI] × 100(3)

Microspheres equivalent to 10 mg of aripiprazole were dissolved in the minimum quantity of dichloromethane and then diluted with the mobile phase: H_2_O pH 3.5: acetonitrile 60:40 (*v*/*v*). The resulting solution was filtered through 0.45 μm filter paper and the filtrate was assayed for ARI using a Shimadzu Prominence HPLC system (Shimadzu Corporation, Kyoto, Japan), consisting of a degasser (Model DGU-20A5), a pump (Model LC-20AD), an automatic sampler (Model SIL-20AC), an ultraviolet–visible variable detector (Model SPD-20A) (λ_max_ = 254 nm) and a thermostatic oven (Model CTO-20AC). A reverse phase C18 column (250 mm × 4.6 mm I.D., 5 µm particle size) was used for chromatographic analysis. The flow rate was adjusted to 1 mL/min and the infusion volume was 20 μL. The chromatograms obtained were processed with the LC Solution software (v1.2, Shimadzu Corporation, Kyoto, Japan). All measurements were conducted in triplicate.

#### 2.5.6. In Vitro Dissolution Test

The MPs (having 10 mg of ARI) were suspended in 2 mL of PBS and inserted in a dialysis tubing cellulose membrane bag (D9402-100FT; Sigma-Aldrich, Steinheim, Germany) with a molecular weight cut-off of 12,000–14,000, which was then sealed and placed into the dissolution basket (Distek Inc., North Brunswick Township, NJ, USA, model 2100C Dissolution Test System), equipped with an automatic sampler (Evolution 4300 Dissolution Sampler). The dissolution studies were performed under sink-conditions, using 400 mL of a phosphate-buffered saline (PBS) solution (pH 7.4), at 50 rpm/37 ± 0.5 °C. The solubility of ARI in PBS was 0.3 mg/mL (measured at 37 °C with the shaking flask method). Samples (2 mL) were withdrawn at predetermined time intervals, filtered and the concentration of ARI was determined using the above described validated HPLC method. Additionally, after the completion of the dissolution experiments the remaining MPs were withdrawn from the dialysis tubes, dried and analyzed for ARI content, using the HPLC method described in Section 2.5.5. All experiments were conducted in triplicate.

In order to evaluate the drug release mechanism, in vitro dissolution results were fitted to the following release kinetics models [33]:

Zero order model: D_t_ = D_0_ + k_0_t (4)

First order model: logD_t_ = logD_0_ + k_1_t/2.303 (5)

Higuchi square root model: D_t_ = D_0_ + k_H_t^1/2^ (6)

Hixon–Crowell model: D_t_^1/3^ = D_0_^1/3^ − k_HC_t (7)

Korsmeyer–Peppas model: D_t_/D_∞_ = D_0_ + k_P_t^n^ (8)

where, D_t_ is the amount of drug released at time t, D_0_ is the initial amount of drug released, D_t_/D_∞_ is the fraction of drug released at time t, k_0_ is the zero-order release constant, k_1_ is the first-order release constant, k_H_ is the Higuchi release constant, k_HC_ is the Hixson–Crowell release rate constant, k_p_ is the Peppas release constant and n is the release exponent respectively.

### 2.6. Statistical Analysis

Statistical significance in the differences of the means was evaluated by using Student’s *t*-test or Dunnett’s test for the single or multiple comparisons of experimental groups, respectively. A difference with a *p*-value (p*) < 0.05 was considered statistically significant.

## 3. Results and Discussion

### 3.1. Evaluation of neat PLA/PBAd Block Copolymers

As stated in the Introduction, the present study attempts to build upon the previously published promising results regarding the thermal and mechanical properties of the newly synthesized PLA/PBAd block copolymers and to evaluate their use as matrix/carriers for the preparation of ARI loaded LAI MPs. In this context, cytotoxicity and enzymatic hydrolysis of the neat copolymers are initially evaluated, since these two features are extremely important before proceeding with the preparation and evaluation of the LAI MPs.

#### 3.1.1. Cytotoxicity Results

In general, polymers or copolymers that will be used to prepare such drug delivery systems should possess low cytotoxicity. Polyesters, based on PLA, poly (glycolic acid) (PGA) and polycaprolactone (PCL) and their copolymers, have been widely used as such biomaterials with a low cytotoxicity profile [34,35,36,37,38,39,40]. However, the cytotoxicity arising from the biodegradation of the newly prepared PLA/PBAd is unknown, and, hence, systematic evaluation is needed in order to verify their safety. The MW of the newly synthesized copolymers (measured by size-exclusion chromatography) varied from 98k–95k, while the polydispersity index (PDI) was below 2.0 in all cases (1.60–1.85) [28].

**Figure 2:**
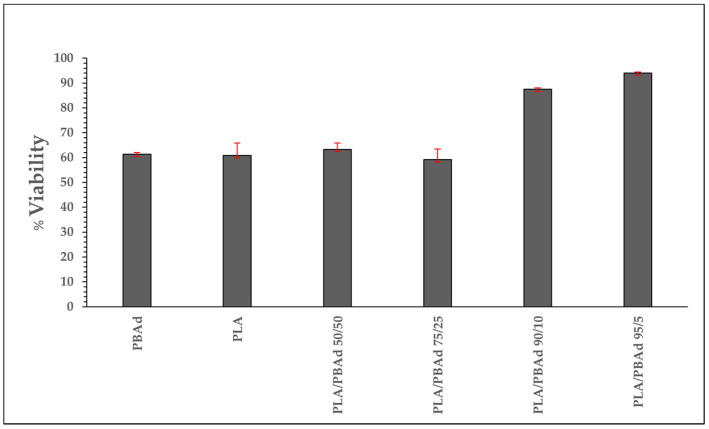
Cytotoxic effect on hAMSCs after incubation with neat PBAd, neat PLA and the newly prepared PLA/PBAd block copolymers.

Figure 2 illustrates the cytotoxicity effect of the prepared block copolymers on hAMSCs, where the *y*-axis shows the reduction of yellow 3-(4,5-dimethythiazol2-yl)-2,5-diphenyl tetrazolium bromide (MTT) by mitochondrial succinate dehydrogenase.

During this study, the MTT, which enters hAMSCs, passes through the mitochondria where it is reduced to formazan. Subsequently, the cells are solubilized and the formazan content is measured spectrophotometrically. Since MTT reduction can only happen in metabolically active cells, the degree of activity is a measure of the cells’ viability. Generally, in order for a material to be classified as toxic a reduction in the measured absorbance should be more than 50% as compared to the control sample. Hence, based on the obtained results, all studied materials can be considered as non-toxic since the max reduction in the measured absorbance was 40%. Specifically, in the case of PLA/PBAd block copolymers the obtained results showed a similar (for PLA/PBAd 50/50 and 75/23 *w*/*w*) or a significantly better (for PLA/PBAd 90/10 and 95/5 *w*/*w*) cytotoxicity profile as compared to the neat PLA, which is considered to be a non-cytotoxic biopolymer. Furthermore, results showed that the metabolic activity of the cancer cell line was dependent on the PLA to PBAd ratio within the copolymer, with samples higher in PLA showing a remarkably lower toxicity. Therefore, based on the MTT assay results it can be said that all prepared copolymers are non-toxic and, hence, are suitable candidates for the preparation drug LAI formulation.

#### 3.1.2. Enzymatic Hydrolysis

In addition to non-toxicity, evaluation of the enzymatic hydrolysis profile of a polymer (or copolymer) is needed in order to clarify whether this material can be used as a LAI matrix/carrier, including, of course, MP based formulations. Generally, enzymatic hydrolysis (i.e., the path to polymer’s degradation) is controlled by various factors related to the structure, the solid and thermal properties, etc. Among them, the mobility of polymer (or copolymer) segments, the crystalline morphology (including spherulite size), the ratio and the balances of hydrophilic/hydrophobic segments, the molecular weight, the T_melt_ and T_g_ are all factors that significantly affect the hydrolysis rate and extent [41,42,43,44,45].

**Figure 3:**
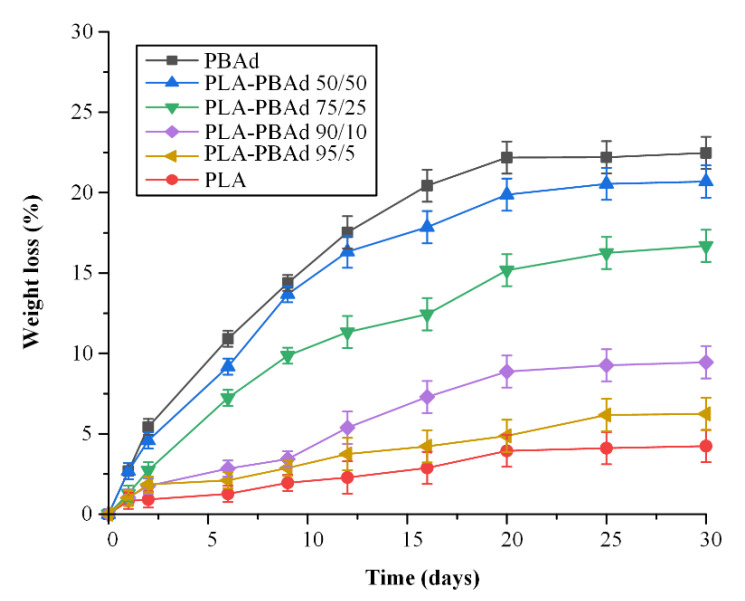
The enzymatic hydrolysis profile measured as % weight loss vs. time plots for the neat PBAd, the neat PLA and the various PLA/PBAd block copolymers.

In the present study, the enzymatic hydrolysis of the raw materials (i.e., the neat PBAd and PLA) and the newly synthesized PLA/PBAd block copolymers were evaluated in solutions containing a mixture of *R. delemar* and *Pseudomonas cepacia* lipases, at 37 °C and pH 7.4. Figure 3 shows the calculated hydrolysis in terms of weight loss vs. time profiles.

In the case of the neat PLA, the results showed an extremely slow enzymatic hydrolysis rate reaching 3% within the first six days of testing. This slow degradation for PLA may be attributed to the polymer’s high hydrophobic nature and to its high degree of crystallinity and its rather high T_melt_ and T_g_ (i.e., 150 °C and 55 °C, respectively [28]). In contrast to PLA, PBAd showed a substantially higher degree of enzymatic hydrolysis. This higher hydrolytic rate is in agreement with previous results [23] and can be attributed to the polymer’s low T_g_ (approximately −55 °C) and T_melt_ onset (40 °C), which allow the polymer’s segments to move around more freely, thus, enabling water to penetrate and hydrolyze the PBAd ester bonds more easily. In the case of PLA/PBAd samples, results showed an increase in the copolymer’s hydrolysis, which was proportional to the PBAd content. Specifically, as the content of PBAd increased, the copolymer’s degradation (measured in terms of weight loss) also increased. Hence, based on the obtained results, it should be noted that the prepared block copolymers also show highly tunable enzymatic hydrolysis characteristics. This is extremely important since, depending on the pharmacological properties of the API, the specific disease features and patients’ individual characteristics, the proposed new biomaterials may be received as a universal solution for tailored drug or patient treatment.

**Figure 4:**
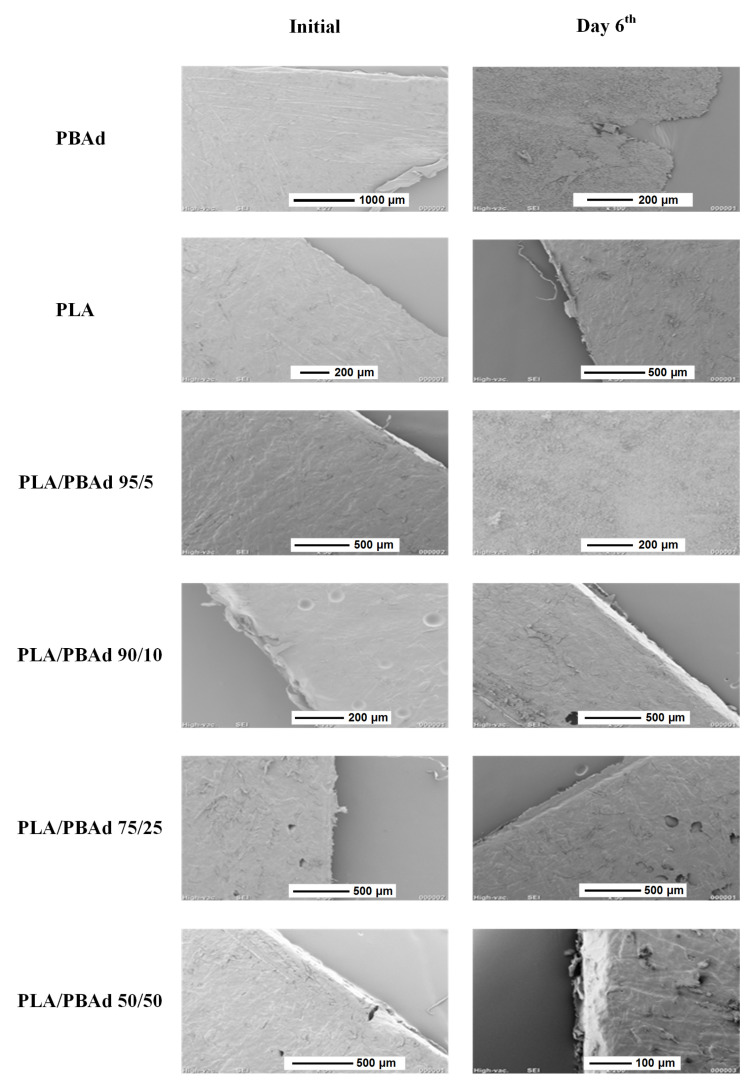
SEM micrographs of the neat PBAd and PLA and the various PLA/PBAd block copolymers during enzymatic hydrolysis at zero time (initial) and after six days.

However, despite the above presented significant findings, regarding the hydrolysis rate and extent of the prepared copolymers, in depth analysis of the degradation process is also needed in order to gain a true insight into the enzymatic biodegradation phenomena. In this context, the morphology of the prepared samples, before and after enzymatic hydrolysis, was evaluated via SEM. The results, presented in Figure 4, showed that the neat PLA remained almost unaffected after six days of testing, while neat PBAd showed an extensive mass degradation, which was dispersed uniformly along the whole surface of the sample. Similarly, the PLA/PBAd copolymers showed increased mass loss, as the content of PBAd increased, while, according to all collected images, it was obvious that the degradation mechanism of the copolymers, during their enzymatic hydrolysis at 37 °C, was related initially to surface erosion. This was also confirmed by SEC measurements after the first six days of study, which showed that molecular weight values remained practically unchanged compared to the initial samples, while weight loss was taking place (Table 2). However, even in this case, hydrolysis is a dynamic procedure. It has been found that the hydrolytic chain cleavage proceeds preferentially in the amorphous regions of polyesters, leading initially to the increase in polymer crystallinity [46]. Due to the interconnections of amorphous fractions, hydrolysis becomes also a bulk erosion process after a period of time.

### 3.2. Evaluation of ARI-loaded MPs

Based on the previously obtained results, the newly synthesized PLA/PBAd block copolymers show a good cytotoxicity profile and highly tunable enzymatic hydrolysis characteristics, features that makes them good candidates as LAI matrix/carriers. Hence, in the following section the preparation of such drug-loaded formulations (in the form of MPs) will be thoroughly evaluated.

#### 3.2.1. MPs Morphology Evaluation Via SEM

**Figure 5:**
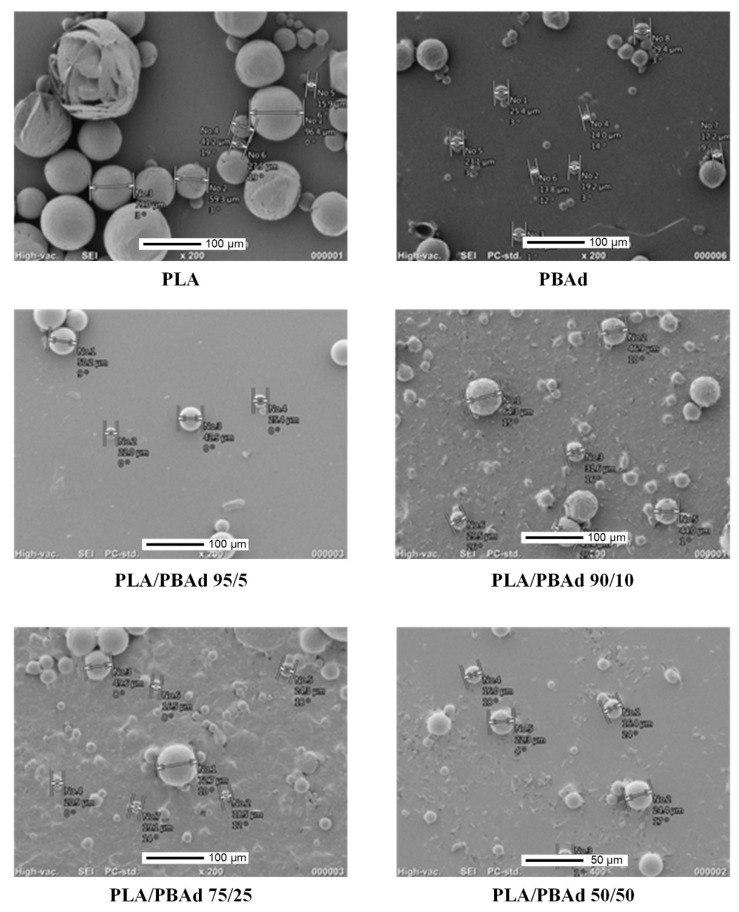
SEM images for the ARI-loaded MPs prepared using PLA, PBAd and the newly synthesized PLA/PBAd block copolymers as the matrix/carriers.

As stated previously, in the present study the newly prepared PLA/PBAd block copolymers (at several PLA to PBAd ratios) were tested as suitable biopolymers for the preparation of ARI LAI MPs. In this set framework, the effect of the PLA and PBAd content on the size and the morphological characteristics of the drug-loaded MPs was initially investigated via SEM. Results in Figure 5 showed the formation of spherical MPs with a smooth exterior surface, while in all cases no particle agglomeration was observed.

Specifically, regarding the MPs prepared with the initial polymeric raw materials (i.e., PLA and PBAd), results showed the formation of significantly larger particles in the case of PLA with more spherical shape and uniform size distribution, while some defects were also observed on the surface of the said MPs. These differences can be attributed to the more hydrophobic nature of PLA (as compared to PBAd), which leads to a better homogenization and consequently more controlled solvent removal processes. Additionally, a significant role also plays the notable differences in the thermal properties of the two tested biopolymers, with PBAd’s lower T_melt_ and T_g_ values enabling the ‘softening’ of the just formed MPs during the solvent removal phase, leading in this way to the formation of smaller drug-loaded spherical MPs (as compared to PLA). In the case of PLA/PBAd, results showed that as the PBAd content increased within the block copolymer the particle size of the obtained MPs decreased. Specifically, the average particle size (measured as d_50_) of the prepared ARI-loaded MPs, measured from at least ten SEM images, was estimated as 58.2 ± 15 µm, 43.3 ± 10 µm, 30.15 ± 10 µm and 18.8 ± 5 µm, for the MPs prepared with PLA/PBAd 95/5, 90/10, 75/25 and 50/50, respectively. Considering the previously published results on the thermal properties of the prepared neat block copolymers [28], where it was found that the melting properties of the two monocomponents (i.e., PLA and PBAd) are retained in the newly prepared biomaterial, the obtained results indicate that the addition of PBAd in the block copolymer chain (and its more hydrophilic nature and lower melting temperature) is responsible for the reduction of the resultant ARI-loaded MP’s size.

#### 3.2.2. MPs Yield, Drug Loading and EE

Table 3 summarizes the yield, drug loading and EE values for the prepared ARI-loaded MPs.

Based on the obtained results, the yield in all MPs ranged from 60.35 to 98.6% with the PLA-PBAd copolymers showing much more improved yields, as compared to the neat PLA and PBAd MPs. Additionally, a closer look at the obtained results revealed that the higher yield values were recorded in the case of MPs containing higher amounts of PBAd (i.e., PLA/PBAd 75/25 and 50/50), indicating that the presence of PBAd in the backchain of the prepared copolymer results in a more efficient (at least in terms of yield productivity) MP’s preparation process. However, in contrary to the previous findings, results regarding MP’s drug loading and EE showed the opposite effect. Specifically, as the content of PBAd increased in the PLA/PBAd copolymer, the resultant ARI-loaded MPs showed lower drug loadings and EE values. This indicates that, contrary to MP’s productivity, the presence of PBAd results in droplets that were harder to solidify, and hence there was much more time for the API molecules to diffuse away from the droplet into the aqueous phase medium, resulting in the preparation of MPs with a lower drug content.

#### 3.2.3. MPs’ Thermal Properties and Physical State Evaluation Via DSC

**Figure 6:**
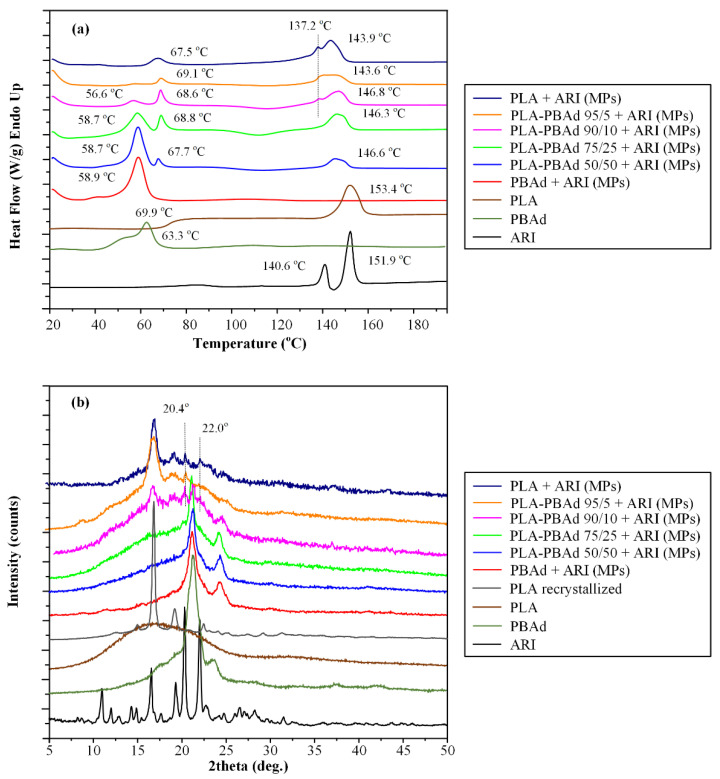
DSC thermograms (**a**) and XRD diffractograms (**b**) of the neat raw materials (i.e., ARI, PLA and PBAd) and the prepared drug-loaded MPs.

The thermal properties and the physical state of the drug-loaded MPs as compared to the neat raw materials were evaluated with the aid of DSC (Figure 6a). In the case of the neat ARI, results showed an initial endothermic peak at 140.6 °C, corresponding to the melting of the ARI form III crystals, followed by a small recrystallization exotherm (at 144 °C) and a second endothermic peak at 151. 9 °C corresponding to the ARI form I crystals melting. These results indicate that the initially used ARI was in the form of polymorph III crystals, while during its melting a phase transition from polymorph III to polymorph I was recorded. This behavior is in agreement with previous studies evaluating the phase transition phenomena occurring during ARI’s DSC heating [47]. Regarding the neat initial polymers, the results in the case of PBAd showed a broad DSC endotherm with a peak at 63.3 °C, corresponding to its melting, while PLA showed a T_g_ transition point at 69.9 °C and a melting endotherm at 153.4 °C, both indicative of its semicrystalline nature.

Looking at the DSC thermograms of the drug-loaded PBAd- and PLA-MPs, similar thermal events were detected, as is in the case of pure (neat) polymers. Specifically, in the case of ARI-PBAd MPs, a broad endothermic peak was recorded at 58.9 °C corresponding to the melting of the crystalline part of the polymer, while for ARI-PLA MPs a T_g_ (with an endothermic overshoot due to the molecules’ relaxation) was recorded at 67.5 °C followed by an endothermic melting peak at 143.9 °C, corresponding to the melting of the PLA. It is important to note that in all thermograms a small drop in the obtained thermal events was recorded (as compared to the neat polymeric raw materials), which is attributed to the presence of the API and the remaining solvents (used for the preparation of the MPs) that act as plasticizers to the whole system.

Additionally, it should be pointed out that in the case of ARI-loaded PBAd MPs, no thermal events were recorded in respect to the API, indicating that probably the drug was amorphously dispersed within the polymeric matrix, although in situ solubilization of the ARI crystals during the DCS heating scan cannot be excluded. On the contrary, results from the DSC thermograms of the drug-loaded PLA MPs, showed a small melting endotherm at 137 °C, which is probably attributed to some of the remaining ARI form III crystals. Similar results were also obtained for the drug-loaded MPs prepared with the newly synthesized PLA/PBAd block copolymers, where the DSC endotherm corresponding to the API melting was decreasing as the PBAd content increased, while at the higher PBAd content used (i.e., PLA/PBAd 75/25 and 50/50) no such API melting peaks were recorded. Hence, based on the DSC results it seems that the presence of PBAd in the PLA/PBAd block copolymers favors the amorphization of the API leading to its complete amorphization in ratios higher that 75/25 *w*/*w* PLA to PBAd.

#### 3.2.4. Physical State Verification Via pXRD

The physical state of the API after the preparation of the drug-loaded MPs was also evaluated via pXRD in order to verify the results suggested by DSC. Figure 6b shows the pXRD diffractograms of the raw materials, the recrystallized neat PLA (after solubilizing in dichloromethane, i.e., the solvent used for the preparation of the MPs) and the respective MPs. In the case of the neat ARI, results showed several sharp pXRD diffractogram peaks at 2θ of 10.9, 16.6, 19.3, 20.3 and 22.0°, which were all characteristic of the ARI form III crystals [48]. In the case of neat PBAd, two characteristic pXRD peaks were recorded at 2θ of 21.1 and 24.2°, which were both located over a broad amorphous halo, indicating that the neat copolymer was semicrystalline in nature. Regarding the neat PLA, two different pXRD patterns were recorded before and after its recrystallization. Specifically, the polymer as received showed a characteristic amorphous halo, indictive of its highly amorphous nature, while upon its recrystallization new crystals were recorded at 2θ positions of 14.8, 16.9, 19.1 and 22.5°, respectively, all of which were also seen in the case of PLA’s melt recrystallization [28]. In regard to the drug-loaded MPs using only PBAd, the recorded pXRD diffractograms showed only the characteristic peaks of the neat copolymer, indicating that the API was amorphously dispersed within the MPs’ matrix/carrier. In contrast, in the case of PLA drug-loaded MPs, in addition to the polymer’s characteristic pXRD pattern, two diffractogram peaks corresponding to the ARI’s form III crystals (i.e., 2θ of 20.4 and 22.0°, respectively) were also recorded, indicating that the API was recrystallized during the formation of the said MPs. Similarly, in the case of MPs prepared with the newly synthesized PLA/PBAd block copolymers having high PBAd content (i.e., 75/25 and 50/50 PLA to PBAd ratio), no ARI characteristic pXRD peaks were recorded, indicating that the API was amorphously dispersed within the said matrix-carriers. On the contrary, in the rest of the samples, i.e., those using PLA/PBAd copolymers with high PLA content, two characteristic ARI form III peaks (although of low intensity) were recorded, indicating that a small portion of the API was recrystallized in these cases. Hence, based on the obtained results, the pXRD analysis verifies the previously presented DSC findings, since the increase in PLA’s content within the newly synthesized PLA/PBAd block copolymers, leads, indeed, to ARI’s recrystallization.

#### 3.2.5. Evaluation of Molecular Interactions

**Figure 7:**
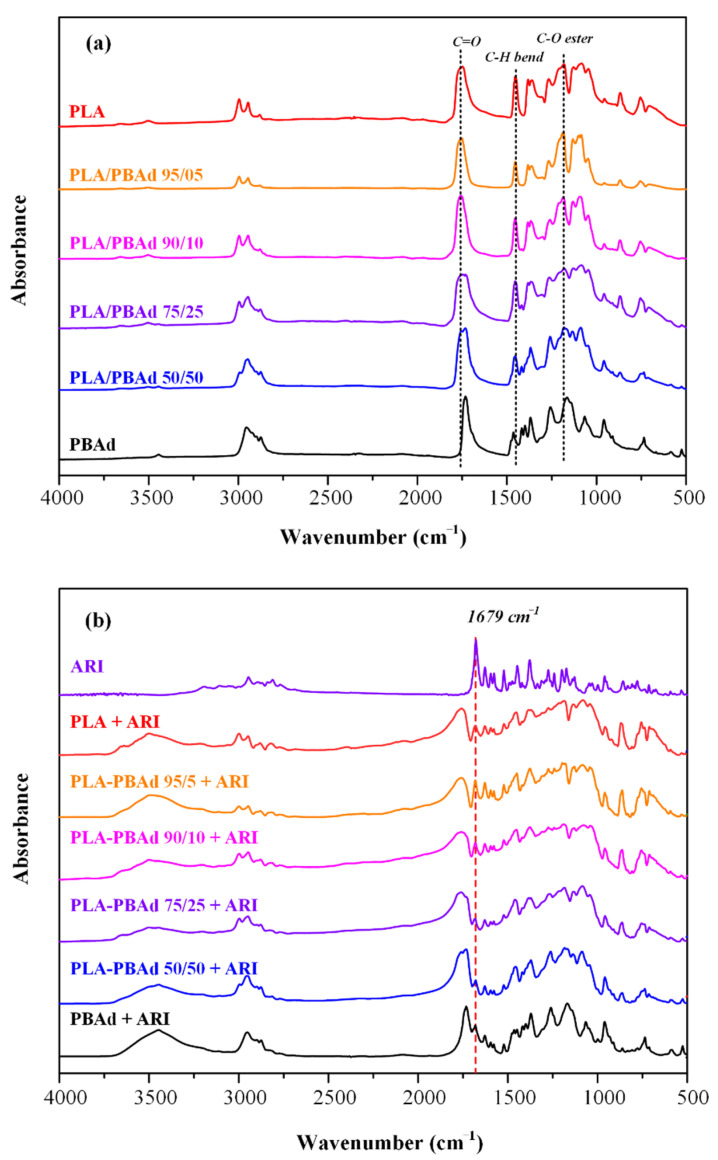
FTIR spectra of: (**a**) the neat PLA, PBAd and the PBA/PBAd copolymers and (**b**) the API and the prepared API-loaded MPs.

In a further step, FTIR spectroscopy was used in order to identify the formation of molecular interactions during the preparation of the drug-loaded MPs. Initially, before proceeding with the FTIR analysis of the MPs, the spectra of the newly prepared PLA/PBAd block copolymers were evaluated (Figure 7a) in an attempt to identify the molecular interactions evolving between the two polymeric components (i.e., PLA and PBAd) during the copolymerization process. Specifically, in the case of PLA the asymmetric and symmetric vibrations of the methylene groups were recorded at 2995 cm^−1^ and 2945 cm^−1^, respectively, while the vibrations of the carbonyl C=O and the C-O-C ester groups were recorded at 1757–1710 cm^−1^ and 1188 cm^−1^. In the case of PBAd, the characteristic absorption peaks of the ester -COO- and the C-O-C appeared at 1735 cm^−1^ and 1100–1300 cm^−1^, respectively, while the peaks located at 1450–1465 cm^−1^ were attributed to the C-H bending vibrations of the methylene and methyl groups. In all spectra the low intensity peaks recorded at 3300–3550 cm^−1^ can be attributed to the presence of -OH end groups. Regarding the newly synthesized copolymers, results showed increased similarities among the recorded spectra. Specifically, in all cases a strong absorption peak at 1730 cm^−1^ was recorded, due to the formation of a new ester bond between the PLA and the PBAd (responsible for the formation of the new block copolymer). Additionally, there were also several peaks in the range of 750–1100 cm^−1^ and 1100–1400 cm^−1^, corresponding to the C-C and C-O vibrations, respectively. Finally, the presence of the methylene groups within the newly synthesized copolymers was also confirmed by the specific FTIR absorption peaks recorded in the region of 2700–3000 cm^−1^.

Moving along with the evaluation of molecular interactions, evolving within the prepared drug-loaded MPs, Figure 7b shows the recorded FTIR spectra of all systems along with the spectrum of the neat API. In regard to ARI, results showed the presence of several characteristic FTIR absorption peaks at 3195 cm^−1^ (corresponding to the NH vibrations), 2949 and 2840 cm^−1^ (attributed to the CH), 1679 cm^−1^, due to C=O, and 1628 cm^−1^, due to C=C, vibrations), while the peaks at 1160 and 1123 cm^−1^ are attributed to the stretching vibration of the single C-O and C-C bonds, respectively. Before proceeding with the analysis of the MPs’ FTIR spectra, it is important to note that in general, polyesters, such as those evaluated in the present study, consist mainly of ester bonds and terminal carboxylic and hydroxyl groups, which can interact, via hydrogen bonding (HB), with the ester groups or the amino groups of ARI and its two chlorine atoms located in the dichlorophenyl part of the molecule. Hence, in order to determine if such interactions exist in the prepared systems, we will focus our analysis on the characteristic peaks recorded in the region of the hydroxyl and carbonyl groups of the FTIR spectrum. Looking at the obtained MPs’ spectra, the hydroxyls of the polyesters in the MPs were recorded at 3480 cm^−1^ and no obvious shifts were apparent amongst the examined systems. Additionally, in the region of carbonyls’ absorption bands, all polyesters show a similar wide peak at 1730 cm^−1^, while next to it (at 1679 cm^−1^) the carbonyl vibration of the pure drug is recorded indicating that the API was successfully encapsulated in the prepared MPs. Finally, since, there are no differences (or shifts/displacements) in the FTIR absorption peaks, it seems that no molecular interactions are taking place between the API and the copolymer.

#### 3.2.6. In Vitro Dissolution Profile

**Figure 8:**
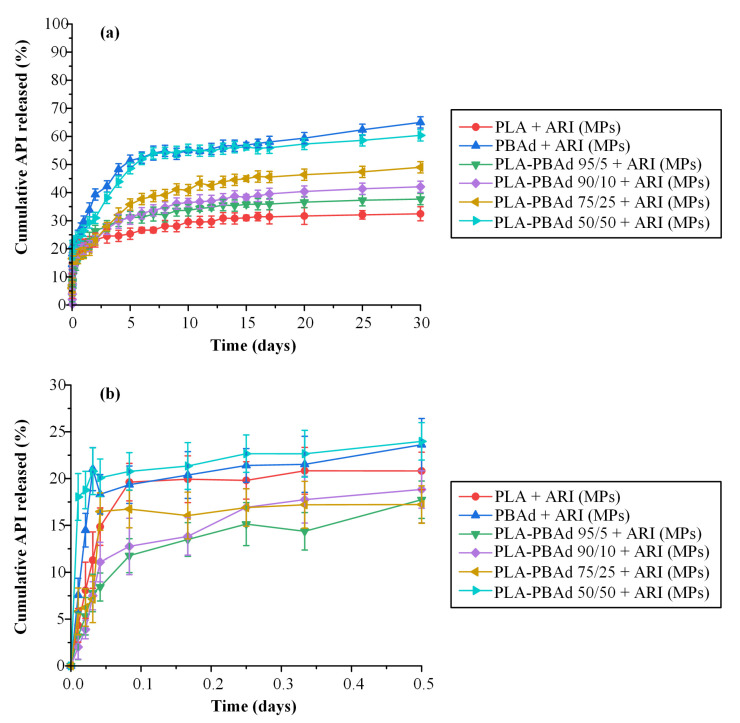
In vitro drug % release vs. time of the encapsulated ARI in PLA/PBAd MPs, during the 30 (**a**) and the 0.5 (**b**) days of the experiment.

In the final step of the present work, the effect of the newly synthesized block copolymers on the in vitro dissolution characteristics of ARI were evaluated. Figure 8a depicts the dissolution profiles of the prepared drug-loaded MPs. The maximum ARI released from the PLA-PBAd MPs ranged from 37.70% (with PLA-PBAd 95/5) to 60.38% (with PLA-PBAd 50/50) indicating a wide distribution, in terms of drug release extent. This can be partially attributed to the amorphization of the drug within the MPs, induced by the presence of the PBAd, and its fine dispersion within the polymeric matrix (confirmed previously by the XRD and DSC results). Additionally, drug assay analysis of the remaining MPs after the completion of the dissolution trials (Table 4) revealed the presence of un-dissolved API still “trapped” within the polymeric structure. This may explain the incomplete delivery of the API observed in all MPs formulations. Lastly, regarding the ARI that was neither recovered from the microparticles nor released, we can postulate that this was probably lost during the withdrawal of the MPs from the dialysis tubes and the drying process conducted before the ARI content analysis. Additionally, we may assume that a small portion of the API may be “lost” due to the drug’s degradation during dissolution, although in order to support/verify this hypothesis ARI solution stability at 37 °C for 30 days has to be performed.

The MPs prepared, using PLA, showed the lowest drug release rate, on contrary to the MPs prepared with PBAd where the maximum release rate was achieved. The rest of the formulations using the newly synthesized PLA-PBAd block copolymers showed increasing API release rate (and extend) as the PBAd content increased. Therefore, it seems that the addition of PBAd to the polymeric matrix significantly improves the hydrolysis rate of PLA and, consequently, the dissolution rate of the encapsulated API. Based on these findings it must be said that the use of the newly synthesized PLA/PBAd block copolymers as matrix/carriers for the preparation of ARI-loaded MPs, results in highly tunable extended release profiles for the API, which may be controlled for up to 30 days. Therefore, under in vivo conditions this could possibly lead to new formulations able to maintain continuous therapeutic levels for an extended time period (>30 days), with no lag-time, and hence, emerge as an alternative long-acting treatment option for the management of chronic diseases.

Looking again back to the obtained dissolution results, it is obvious that ARI’s dissolution from the prepared MPs followed a biphasic release profile in all cases. Specifically, after an initial burst phase (Figure 8b) attributed to the active substance present on the surface of the MPs, a fast release phase was observed for up to approximately five (5) days, followed by a slower release phase for the remaining twenty-five (25) days. Keeping in mind that drug release from such MPs is mainly controlled by the interplay between API’s diffusion from the polymeric matrices and polyester’s erosion/degradation behavior, it can be assumed that in both phases (i.e., the fast and the slow) these two different mechanisms have a different impact. Therefore, in an attempt to identify the differences prevailing in each release phase, the obtained dissolution data were fitted in the various kinetic models described in Section 2.5.6. The goodness of fit (expressed by the correlation coefficient, R^2^) and the k-constants for each model are summarized in Table 5.

Looking at the obtained results, in the case of the initial release phase (i.e., up to five days) the higher R^2^ values for all samples were obtained for the Korsmeyer–Peppas equation, indicating that the said model is more suitable to describe the obtained dissolution data. In general, the Korsmeyer–Peppas model is able to describe the several mechanisms that simultaneously control the dissolution behavior in such systems, by the use of the exponent *n*. Specifically, *n* values below 0.5 suggest that the drug diffuses through the matrix and is released with a quasi-Fickian diffusion mechanism, while values between 0.5 and 1 indicate an anomalous, non-Fickian, drug diffusion and values above 1 suggest a non-Fickian, Case II, release kinetics mechanism [33]. Based on the obtained Korsmeyer–Peppas fitting results, the exponent *n* in the initial fast release phase was below 0.5 in all cases (i.e., *n*_(PLA)_ = 0.088, *n*_(PBAd)_ = 0.337, *n*_(PLA/PBAd 95/5)_ = 0.268, *n*_(PLA/PBAd 90/10)_ = 0.256, *n*_(PLA/PBAd 75/25)_ = 0.366 and *n*_(PLA/PBAd 50/50)_ = 0.348) indicating that the drug released from all prepared MPs in the first five days was diffusion controlled. Interestingly, in the case of the slow-release phase (i.e., starting from the 6^th^ day and lasting up to 30 days) the fitting results in Table 5 showed that the release of the API from all prepared MPs followed a zero-order release mechanism. This is also verified by the *n* exponent of the Korsmeyer–Peppas model fitting, where, in all cases, was approximately one (i.e., *n*_(PLA)_ = 0.917, *n*_(PBAd)_ = 1.057, n_(PLA/PBAd 95/5)_ = 0.920, *n*_(PLA/PBAd 90/10)_ = 0.989, *n*_(PLA/PBAd 75/25)_ = 1.032 and *n*_(PLA/PBAd 50/50)_ = 1.066). Hence, it seems that in the latter stage of API’s dissolution the initially diffusion-controlled phase is compensated by the simultaneous matrix swelling (due to the polyester’s wetting) and a small portion of matrix erosion (due to the polyester’s hydrolytic degradation) leading in this way to a ‘balanced’ zero-order release profile, which is essential in achieving stable in vivo pharmacokinetic behavior.

##### Morphology Evaluation after Dissolution Studies

**Figure 9:**
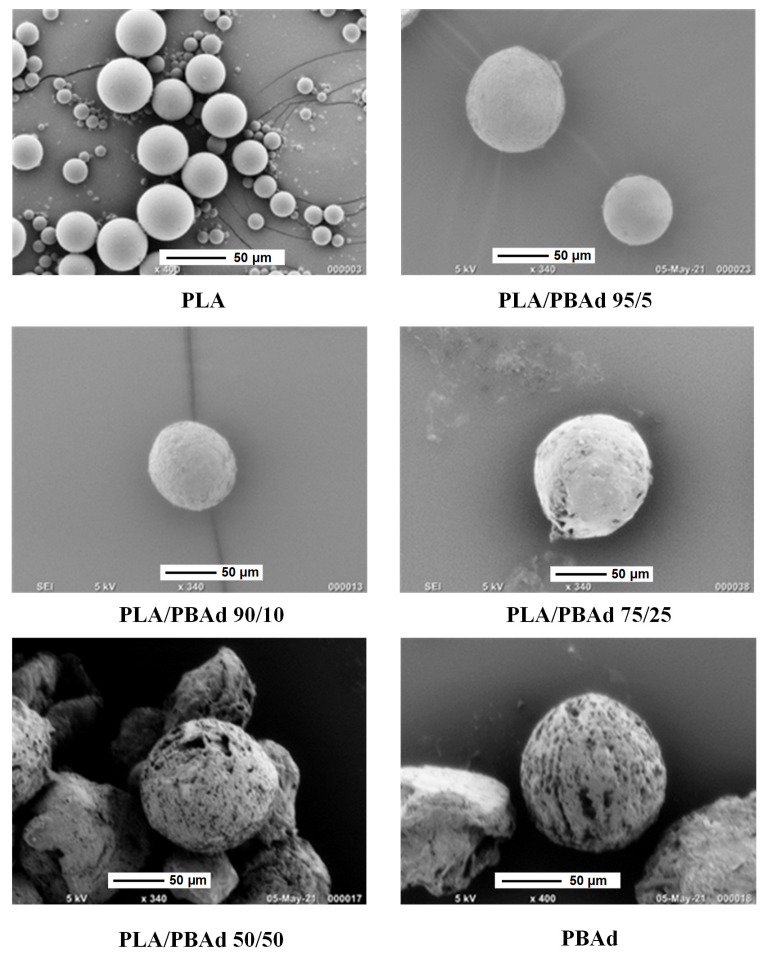
SEM images of the polyester erosion process after 30 days of dissolution.

In a further step, in order to examine the process of polyester degradation/erosion during dissolution and to correlate this with the enzymatic hydrolysis results presented in Section 3.1.2, SEM images were taken after the completion of the test (Figure 9). As evidenced, in the case of neat PLA and the two polyesters containing only a small amount of PBAd, namely PLA/PBAd 95/5 and 90/10, the surface and shape of the prepared microspheres remained practically unchanged (Figure 9). On the other hand, neat PBAd and the polymeric matrices containing high PBAd load (i.e., PLA/PBAd 75/25 and PLA/PBAd 50/50) demonstrated some clear evidence of surface erosion, presumably due to polyester hydrolysis or drug dissolution. From these images, we can thus conclude that the amount of PBAd bares a crucial role to the extent of polyester degradation and consequently the drug release rates, which is in accordance to previously discussed results from neat polymer enzymatic hydrolysis studies.

##### A Mechanistic Release Model

Finally, since the so-called “standard” dissolution release models used in the literature and herein (see Equations (4)–(8)) present several limitations related to the assumptions made for their implementation (for details please see Reference [33]), new, more sophisticated models were also tested for modeling the obtained results.

In general, there are two types of models to describe a physicochemical process such as the drug’s dissolution. The first kind is the so-called empirical models. The physical content of these models is limited. Some of them are just equations used to describe appropriately a large amount of experimental data and some of them have a kind of qualitative information of the physical mechanism that is responsible for the process evolution. The second type of models are the so-called mechanistic models. These models include information of the underlying mechanism and in addition they can consider several mechanisms acting simultaneously. After considering exhaustively the whole toolbox of existing empirical models to describe the present data, an attempt to construct a mechanistic model of the present release process was made.

Looking closely at the form of the release data, the performance of the “standard” release models and the data for hydrolysis evolution of the polymer matrices, the following scenario appears: There is an initial fast release phase that can be partially attributed to the presence of API probably in the form of a thin film layer located on, or near, the surface of the MPs. It is not clear if the mechanism of this layer release is diffusion or matrix erosion since both are equally probable. The second release phase (which is slower) is mostly controlled by Fickian diffusion, although a small erosion contribution is also there (verified by the SEM images presented in Figure 9). Assuming that a fraction of the drug in the polymer is free to move and its motion occurs through the diffusion mechanism and that the shape of the particles is approximately spherical (verified by SEM that is imaged in Figure 5), the transient partial differential equation of diffusion is probably the best model to describe the dissolution behavior of the API [49]. However, in this case a very simple exponential form, called the linear driving force approximation, can be also used to model the obtained results [50]. This same approximation was also used in the present study for modeling the dissolution kinetics of the API located on the surface layer, despite its unknown release mechanism. Finally, there is a fraction of drug immobilized in the polymer matrix. This fraction can be released only through matrix erosion (due to polyester hydrolysis). In the absence of any other information a zero order release dynamics model will be assessed for this fraction. It should be pointed out that for the limited extent of hydrolysis observed here, this approximation is quite realistic. By summarizing the above arguments, the released drug fraction evolution can be approximated by the following (uniformly valid in time) expression:

(9) $$C_{r}=\varphi_{1}(1-\exp(-k_{1}t))+\varphi_{2}(1-\exp(-k_{2}t))+k_{3}t$$

where C_r_ is the cumulative API released (%), φ_1_ and k_1_ are the percentage of drug in the excess layer and the corresponding kinetic constant respectively, φ_2_ and k_2_ are the percentage of mobile drug and the corresponding kinetic constant respectively and k_3_ is the kinetic constant of the erosion process. It is noted that the linear superposition of diffusion- and erosion-induced release is allowed only because the erosion extent is small.

**Figure 10:**
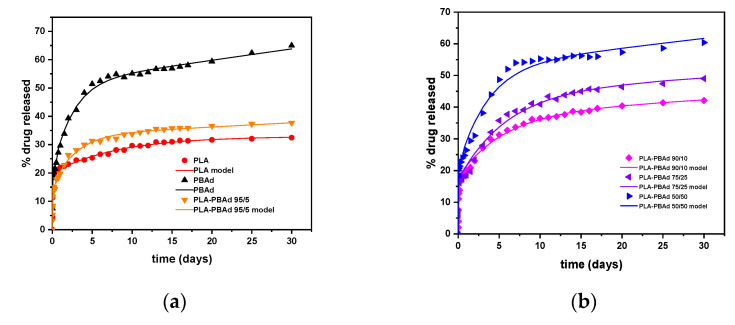

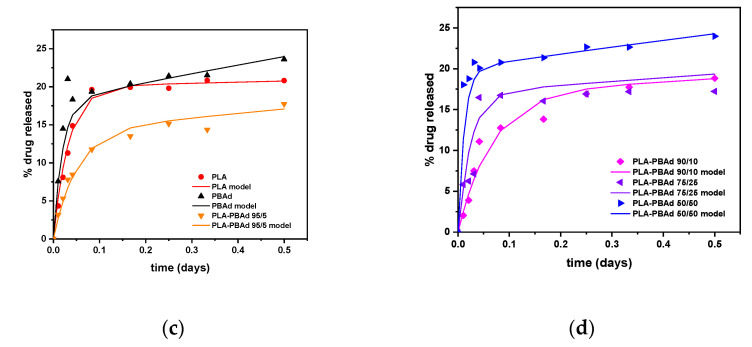
Comparison of experimental release data (symbols) to the mechanistic model-based Equation (9) (continuous lines). The presentation is made in two-time scales just for clarity: (**a**,**b**) 0–30 days and (**c**,**d**) 0–0.5 days.

Figure 10 shows the comparison between the predicted and the experimentally derived points after fitting to Equation (9).

The R^2^ factor was larger than 0.99 in all cases except for composites 75/25 and 50/50 for which it was 0.98 and 0.99, respectively (probably due to a more complicated release scenario than the one described by Equation (9)). Nevertheless, and despite this small pitfall, the mechanistic model proposed herein is still more efficient compared to the “standard” empirical model tested previously, since it is able to model the dissolution profile of the API within the whole-time domain of the test (i.e., both release phases simultaneously). The values of the fitting parameter according to Equation (9) are presented in Table 6.

According to the obtained results, the percentage of drug in the excess layer was 20% and the corresponding parameters φ_1_ and k_2_ did not show any systematic correlation to the copolymer matrix composition. This is expected to be the case for a rather random procedure of accumulation of drug in the surface layer. The fraction of the mobile drug appears to increase consistently from 13% (for PLA) to 32% (for PBAd), while the corresponding kinetic constant k_2_ appeared also to increase in the same order (with the exception of the 95/5 composite). Finally, the erosion constant k_3_ increased as the content of PBAd increased, which is in agreement with the hydrolysis rates evaluation presented previously.

The diffusion coefficient, D, of the mobile drug presented also in Table 6 was calculated based on the following equation [50]:

D = k_2_r^2^/15 (10)

where r is the radius of the particles (presented in Table 6). Based on the obtained results, the range of values corresponding to D consisted of the drug diffusion within the polymer matrix and is in agreement with the results describing the second (and slower) release phase (depicted by k_2_ constant). However, no such relation was proven in the case of the k_1_ constant, since the characteristic length of diffusion in the first fast release phase is unknown. So, it can be said that the release of the drug’s initial fraction (i.e., φ_1_) may be either from the fast erosion of the very thin API layer located on the surface of the MPs or due to the fast initial API diffusion from this surface layer.

## 4. Conclusions

In the present study PLA/PBAd-based ARI-loaded LAI MPs were successfully prepared for the first time. Results regarding the highly tunable enzymatic hydrolysis profile and the low cytotoxicity of the new copolymers, amplified the previously made suggestions that these new copolymers can be considered as a quite promising candidate for the preparation of drug sustained release formulations. Evaluation in terms of morphological characteristics (via SEM), productivity (in terms of MPs’ yield) and drug loading also showed extremely promising results. Physicochemical analysis of the prepared formulations revealed the amorphous API dispersion with increasing PBAd content, while no specific molecular interactions between the drug and the polyesters were recorded, based on FTIR spectroscopy. Lastly, in terms of the in vitro dissolution profile, results suggested that the newly synthesized PLA/PBAd block copolymers can successfully control the release rate and extent of the API’s release from the prepared MPs, indicating that, probably, under in vivo conditions their use may lead to new formulations that will be able to maintain a continuous therapeutic level for an extended time period (>30 days), with reduced lag-time, as compared to the currently marketed ARI LAI product.

## Figures and Tables

**Table 1:** Values of interest for the synthesized copolymers: molecular weight values, weight averaged (M_w_) and number averaged (M_n_), and polydispersity index (PDI), estimated by SEC, and crystallization, melting and glass transition temperatures (T_c_, T_m_ and T_g_ accordingly), estimated by DSC.

Technique	SEC			DSC					
				PBAd			PLA		
Sample	*M*_w_ (g/mol)	*M*_n_ (g/mol)	PDI	*T*_c_ (°C)	*T*_g_ (°C)	*T*_m_ (°C)	*T*_c_ (°C)	*T*_g_ (°C)	*T*_m_ (°C)
PLA	130k	73k	1.79	-	-	-	-	55	149/155
PLA/PBAd 95/05	98k	61k	1.60	21/32	-	53	-	53	148/155
PLA/PBAd 90/10	97k	59k	1.65	15/32	-	52	-	54	148/155
PLA/PBAd 75/25	95k	57k	1.66	2/31	–64	52/55	-	54	147/154
PLA/PBAd 50/50	98k	57k	1.73	28	–60	52/55	-	54	148/154
PBAd	90k	49k	1.85	29	–62	55	-	-	-

**Table 2:** SEC estimated molecular weight values, M_w_ and M_n_, and % weight loss, after six days of enzymatic hydrolysis.

Sample	Mw (g/mol)	Mn (g/mol)	% Weight Loss
PLA	129.7k	72.8k	1.267
PLA/PBAd 95/05	97.8k	60.8k	2.109
PLA/PBAd 90/10	97.1k	58.6k	2.837
PLA/PBAd 75/25	93.9k	56.9k	7.230
PLA/PBAd 50/50	97.9k	57.1k	9.178
PBAd	88.9k	47.9k	10.912

**Table 3:** Yield, drug loading and EE of the prepared ARI MPs.

Sample	Average Particle Size (d_50_) (μm)	Yield (%)	Drug Loading (%)	EE (%)
PLA	56.3 ± 15	77.73 ± 2.84	16.35 ± 1.75	42.73 ± 2.08
PLA/PBAd 95/5	58.2 ± 15	89.32 ± 2.03	14.56 ± 1.86	44.84 ± 2.87
PLA/PBAd 90/10	43.3 ± 10	92.51 ± 2.48	13.19 ± 2.83	38.17 ± 3.54
PLA/PBAd 75/25	30.2 ± 10	98.60 ± 1.38	12.48 ± 2.16	39.78 ± 2.86
PLA/PBAd 50/50	18.8 ± 5	97.40 ± 1.24	11.30 ± 3.14	32.67 ± 3.07
PBAd	21.3 ± 5	60.35 ± 3.68	17.48 ± 2.47	36.52 ± 4.23

**Table 4:** % Aripiprazole drug remained in the MPs at the end of the in vitro release study.

Sample	Remained ARI (%)
PLA	66.28 ± 0.23
PLA/PBAd 95/5	60.08 ± 2.12
PLA/PBAd 90/10	58.42 ± 1.54
PLA/PBAd 75/25	50.63 ± 2.03
PLA/PBAd 50/50	38.94 ± 1.78
PBAd	34.78 ± 2.27

**Table 5:** Dissolution data model fitting results for the employed drug release kinetic models.

Release Fitting Model	PLA		PBAd		PLA/PBAd							
					95/5		90/10		75/25		5050	
	R^2^	k-Constant	R^2^	k-Constant	R^2^	k-Constant	R^2^	k-Constant	R^2^	k-Constant	R^2^	k-Constant
Fast-release phase												
Zero order	0.60	2.77 d^−1^	0.77	8.03 d^−1^	0.68	4.61 d^−1^	0.67	4.32 d^−1^	0.82	5.54 d^−1^	0.80	7.43 d^−1^
First order	<0.01	0.09 d^−1^	0.58	0.20 d^−1^	0.16	0.11 d^−1^	0.12	0.10 d^−1^	0.54	0.11 d^−1^	0.57	0.17d^−1^
Higuchi	0.31	14.84 d^−1^	0.93	25.45 d^−1/2^	0.83	16.45 d^−1/2^	0.81	16.03 d^−1/2^	0.94	16.67 d^−1/2^	0.93	22.95 d^−1/2^
Hixson–Crowell	<0.01	0.05 d^−1^	0.69	0.06 d^−1^	0.08	0.03 d^−1^	0.04	0.03 d^−1^	0.48	0.03 d^−1^	0.49	0.05 d^−1^
Korsmeyer–Peppas	0.99	21.90 d^−n^	0.99	29.93 d^−n^	0.99	20.66 d^−n^	0.98	20.35 d^−n^	0.98	19.06 d^−n^	0.98	26.70 d^−n^
Slow-release phase												
Zero order	0.77	4.20 d^−1^	0.98	3.91 d^−1^	0.84	3.98 d^−1^	0.89	4.02 d^−1^	0.87	4.08 d^−1^	0.93	3.42 d^−1^
First order	0.69	0.07 d^−1^	0.71	0.03 d^−1^	0.75	0.06 d^−1^	0.77	0.06 d^−1^	0.74	0.06 d^−1^	0.80	0.04 d^−1^
Higuchi	0.60	16.64 d^−1/2^	0.53	10.58 d^−1/2^	0.66	16.08 d^−1/2^	0.66	15.21 d^−1/2^	0.62	14.60 d^−1/2^	0.66	12.79 d^−1/2^
Hixson–Crowell	0.74	0.02 d^−1^	0.76	0.01 d^−1^	0.81	0.02 d^−1^	0.83	0.02 d^−1^	0.79	0.01 d^−1^	0.85	0.01 d^−1^
Korsmeyer–Peppas	0.76	5.21 d^−n^	0.98	0.61 d^−n^	0.83	4.99 d^−n^	0.88	3.89 d^−n^	0.85	3.31 d^−n^	0.93	2.62 d^−n^

**Table 6:** Parameters derived by fitting Equation (9) to the experimental drug release data.

Material	φ_1_	φ_2_	k_1_ (d^−1^)	k_2_ (d^−1^)	k_3_ (d^−1^)	D × 10^17^ (m^2^/s)
PLA	20	13	30	0.12	0	7.4
PLA-PBAd 95/5	14.5	18.25	20	0.36	0.15	23.5
PLA-PBAd 90/10	17.5	20	15	0.18	0.15	6.5
PLA-PBAd 75/25	16.25	28	40	0.17	0.15	3
PLA-PBAd 50/50	20.5	31.7	80	0.28	0.3	1.9
PBAd	19	32	50	0.4	0.4	3.5
